# Editorial: Safety in close human-robot interaction

**DOI:** 10.3389/frobt.2023.1288713

**Published:** 2023-10-20

**Authors:** Marcello Valori, Gerdienke Prange-Lasonder, José Saenz, Roland Behrens, Catherine Bidard, Eric Lucet, Irene Fassi

**Affiliations:** ^1^ Institute of Intelligent Industrial Technologies and Systems for Advanced Manufacturing, National Research Council of Italy, Milan, Italy; ^2^ Roessingh Research and Development, Enschede, Netherlands; ^3^ Department of Biomechanical Engineering, University of Twente, Enschede, Netherlands; ^4^ Fraunhofer IFF, Magdeburg, Germany; ^5^ Université Paris-Saclay, CEA, LIST, Palaiseau, France

**Keywords:** human-robot collaboration, human-robot interaction, safety, ergonomics, service robots, industrial robots, medical robots

The concept of Human-Robot Collaboration (HRC) has changed the industrial work paradigms as a key enabling technology of the Industry 4.0 transition. In the next industrial epoch, HRC potentialities will become even more crucial as the human-centric perspective requires optimal work sharing between workers and robots. At the same time, robots are spreading even outside the industrial environment in a variety of activities, assisting and interacting with humans in different ways and at different levels. In general, Human-Robot Interaction (HRI) is evolving, becoming closer and closer and often characterized by physical interaction, thus paving the way for a new implementation era for a wide range of robots and robotic devices.

With this perspective, new safety challenges are to be addressed to avoid injuries caused by human-robot collisions, musculoskeletal damage, or excessive mental load created by a potentially inappropriate physical interaction with robotic devices. In relation to this, we also see an increase in activities to update standards or create new ones that support the design and assessment of new applications featuring industrial, service, or medical robots.

In parallel, the research community is investigating a variety of technologies that enable the increase of safety in “close” HRI, including innovative approaches and perspectives for risk assessment, verification, and validation of applications in this field. In the industrial environment, contact prevention without barriers and fences usually relies on the implementation of advanced sensing systems and control strategies; contacts must be detected—possibly predicted—and mitigated by inherent safety features and protection measures. The coordination of humans and robots must comply with ergonomic principles to reduce physical and mental stress and support synergic collaboration.

These issues are analog to those affecting most service robots designed on a general basis to interact with humans. As an example, robots deployed in public spaces shall often perform autonomous navigation, with the capabilities of safely bypassing obstacles and humans. Looking at the medical and healthcare domain, several robotic implementations are mature and well-established while even more are still being developed and introduced to clinical practice: “RACA” (rehabilitation, assessment, compensation, and alleviation) robots, surgical robots, bionic prostheses, and caregiver robots. It is worth pointing out that safety in human-robot interaction becomes even more critical when involving non-expert users, let alone more vulnerable users with physical or cognitive limitations, potentially representing a barrier to full performance in robot implementations. More sophisticated and dynamic ways of measuring safety-related behavior are needed to allow the use of the full potential of HRC. This should not be limited to physical dimensions and robot-centered measures but must also address physiological aspects of the user’s behavior, including user perceptions of the interaction between humans and robots, especially in the case of non-expert or impaired users.

This Research Topic includes three papers, each one related to a different application domain–industrial, service (public spaces), and medical (rehabilitation), respectively–which are hereafter briefly described.

Walter et al. deal with speed and separation monitoring (SSM), which is the HRC mode in which contact between humans and robots is prevented by evaluating the safety distance necessary to ensure a safe robot stop at any time. A too-conservative evaluation of such a distance may cause suboptimal task execution, with a consequent increase in the execution time. The authors propose a voxel-based methodology to calculate the “safety volumes” of both robots and humans dynamically. The former is given by the space occupied by the robot, increased by the volume potentially swept in the upcoming movements, and the worst-case space necessary for braking. The operator-related safety space generation is based on camera acquisition, by segmenting the human volume and adding uncertainty and safety margins. Upon an overlap of the two volumes generated this way, robot stopping must be initiated.

Tan et al. present a survey conducted in a primary care clinic in Singapore after the introduction of a service robot, with the objective of determining the acceptability, perceptions of safety, and concerns regarding the robot. The questionnaire survey was distributed to patients, their accompanying persons, and the clinic healthcare workers. The robotic unit consists of a mobile platform equipped with a thermal camera to monitor the temperature of the interfacing persons, a camera to detect proper mask wearing, a front screen for user interface, and a UV-based disinfection dome. Before the deployment and survey, the safety performance of the robot was evaluated with regard to its speed control, obstacle avoidance, and disinfection capability. The results of the survey reveal a general approval of the functionalities provided by the robot, especially from the perspective of reducing infection risks. However, safety related to human-robot coexistence turned out to be a primary concern of the participants, suggesting that further safety-related functionalities should be included and, possibly, that an information campaign should be performed before robot deployment.

Ranzani et al. discuss the monitoring of patient muscle tone during robot-assisted rehabilitation, aimed at improving safety during intensive treatments. A two-degree freedom haptic device is used to enable patients to perform grasp and pro/supination exercises, while the device provides force feedback, complemented by a virtual reality interface, showing the objects the patient is interacting with. A specific protocol is proposed in a pilot study to detect muscle tone during the execution of an object identification exercise to both subjects with and without spasticity in the lower arm and hand muscles. With a sampling time of a few minutes, the force peaks induced by external perturbations and stiffness exerted by the fingertips are estimated. Besides the numerical results, the protocol was completed by all the participants without adverse events, and the differences between the subject groups were successfully identified.

This Research Topic aims to provide evidence that although the field regarding close HRI (in the sense of shared space between robot and human) is still young, research efforts are needed - and are actually increasing - to advance HRI by solving the potential safety-related barriers for full exploitation of the technological potential.

